# Patient similarity analytics for explainable clinical risk prediction

**DOI:** 10.1186/s12911-021-01566-y

**Published:** 2021-07-01

**Authors:** Hao Sen Andrew Fang, Ngiap Chuan Tan, Wei Ying Tan, Ronald Wihal Oei, Mong Li Lee, Wynne Hsu

**Affiliations:** 1 SingHealth Polyclinics, SingHealth, 167, Jalan Bukit Merah, Connection One, Tower 5, #15-10, Singapore, P.O. 150167, Singapore; 2 Family Medicine Academic Clinical Programme, SingHealth-Duke NUS Academic Medical Centre, Singapore, Singapore; 3 Institute of Data Science, National University of Singapore, Singapore, Singapore; 4 School of Computing, National University of Singapore, Singapore, Singapore

**Keywords:** Patient similarity, Prediction models, Explainable artificial intelligence, Interpretable, Clinical decision support tool

## Abstract

**Background:**

Clinical risk prediction models (CRPMs) use patient characteristics to estimate the probability of having or developing a particular disease and/or outcome. While CRPMs are gaining in popularity, they have yet to be widely adopted in clinical practice. The lack of explainability and interpretability has limited their utility. Explainability is the extent of which a model’s prediction process can be described. Interpretability is the degree to which a user can understand the predictions made by a model.

**Methods:**

The study aimed to demonstrate utility of patient similarity analytics in developing an explainable and interpretable CRPM. Data was extracted from the electronic medical records of patients with type-2 diabetes mellitus, hypertension and dyslipidaemia in a Singapore public primary care clinic. We used modified K-nearest neighbour which incorporated expert input, to develop a patient similarity model on this real-world training dataset (n = 7,041) and validated it on a testing dataset (n = 3,018). The results were compared using logistic regression, random forest (RF) and support vector machine (SVM) models from the same dataset. The patient similarity model was then implemented in a prototype system to demonstrate the identification, explainability and interpretability of similar patients and the prediction process.

**Results:**

The patient similarity model (AUROC = 0.718) was comparable to the logistic regression (AUROC = 0.695), RF (AUROC = 0.764) and SVM models (AUROC = 0.766). We packaged the patient similarity model in a prototype web application. A proof of concept demonstrated how the application provided both quantitative and qualitative information, in the form of patient narratives. This information was used to better inform and influence clinical decision-making, such as getting a patient to agree to start insulin therapy.

**Conclusions:**

Patient similarity analytics is a feasible approach to develop an explainable and interpretable CRPM. While the approach is generalizable, it can be used to develop locally relevant information, based on the database it searches. Ultimately, such an approach can generate a more informative CRPMs which can be deployed as part of clinical decision support tools to better facilitate shared decision-making in clinical practice.

**Supplementary Information:**

The online version contains supplementary material available at 10.1186/s12911-021-01566-y.

## Introduction

Clinical risk prediction models (CRPM) are designed to assist healthcare professionals in making better clinical decisions [1]. In general, CRPMs use patient characteristics to estimate the probability about having (or developing) a particular disease (or outcome) [2]. As healthcare knowledge continues to expand and outstrip human cognitive capacity, CRPM have gained popularity as they offer a scalable way to consolidate growing volumes of data and information complexity to support clinical decision-making [3]. Such CRPMs range from predicting hospital readmissions, to various types of cancers, and more recently COVID-19 [4–7].

Despite their proliferation, CRPMs have yet to be adopted in clinical practice on a larger scale [8, 9]. While concerns regarding rigour in development and validation of CRPMs are being addressed by established guidelines, attention is shifting toward improving their explainability and interpretability [9–19]. Explainability is defined as the extent of which a model’s prediction process can be described, while interpretability is defined as the degree to which a user can understand the predictions made by a model [20–22].

Recently, patient similarity analytics has become a popular technique for CRPM development [23]. The underlying concept is to identify similar patients to a patient of interest, and use them as a clinically meaningful subgroup to derive more precise prognostic information [24], and has also been shown to improve prediction accuracy [25, 26]. One advantage of this technique is that it is able to display the similar patients that it uses to make the predictions. This increases the transparency in the prediction process, thus improving model explainability. With the similar patients, case-based narrative can thus be crafted around the predictions to enhance their interpretability.

## Methods

### Study aim

This study aims to demonstrate the deployment of patient similarity analytics to develop an explainable and interpretable CRPM using an electronic medical records derived dataset of patients with type-2 diabetes mellitus (D), hypertension (H) and dyslipidaemia (L) and their DHL-related complications in primary care.

### Data description

This study was conducted using a real-world dataset consisting of de-identified electronic medical records of patients who visited a polyclinic in south-eastern Singapore. This polyclinic manages about 450 to 500 patient attendances daily during office hours and serves about 350,000 multi-ethnic Asians living in the district. About one-third of patients who attend the polyclinic are aged 65 and above. For the purpose for this study, patients who visited for any of the DHL conditions during the period of April 1, 2014 to March 31, 2015 were included in the dataset. Their demographic characteristics, disease history, laboratory test results and prescribed medications data were extracted over a 10-year period from April 1, 2009 to March 31, 2019. Ethics board approval was obtained before the conduct of this study (SingHealth Centralized Institutional Review Board Reference Number: 2019/2604).

### Data definitions

The first visit of each patient during the period of April 1, 2014 to March 31, 2015 was denoted as the base visit. This was the index visit used to provide a cross-sectional representation of each patient’s disease status, including years with disease, medications, and complications. The look-back period (April 1, 2009 to March 31, 2014) was used to obtain the DHL disease history, while the look-forward period (April 1, 2014 to March 31, 2019) was used to obtain data on DHL complication onset.

Patients’ onset of any one or combination of DHL conditions was their earliest visit with a pre-defined set of International Classification of Disease 9th or 10th revision (ICD) codes, or relevant medications (Table 2) in the look-back period. Patients with type-2 diabetes mellitus (D) were defined by ICD codes 250.90, 250.40, 250.80, E11.9, E11.21, E11.22, E14.31, E14.73 and E11.40, or if they were on insulin or other oral anti-diabetic medications. Patients with essential hypertension (H) were defined by ICD codes 401.1, 796.2, I10, or if they were being treated with any one or more anti-hypertensive medications. Patients with dyslipidemia (L) were defined by ICD codes 272.0, E78.5, or if they were taking prescribed lipid-lowering medication(s).

Patients were deemed to have DHL-related complications if their visit history in both the look-back and look-forward periods contained predefined set of ICD codes in Table 1. In addition to the ICD codes, patients were considered to have an eye complication if they had a diabetic referrable finding on eye examination and/or were on follow-up with an eye specialist. Patients were deemed to suffer from a foot complication if they have been flagged as high risk for foot ulcer during an examination and/or were on follow-up with a podiatrist or vascular surgeon. Patients were also deemed to have kidney complication if they had estimated glomerular filtration rate < 60 ml/min/1.73m^2^ (based on CKD-EPI [Chronic Kidney Disease Epidemiology Collaboration] equation); and macrovascular complication if they had been prescribed the following antiplatelet medications: aspirin, clopidogrel, dipyridamole or ticagrelor. [27].

**Table 1:** International Classification of Diseases 10 codes for eye, foot, kidney and macrovascular complications

Complication	ICD codes
Eye	E1431, 3620
Foot	E1140, E1473, I739, 4439
Kidney	E1122, 25,040, N183, N184, N185, 5859, 585
Macrovascular	I249, I259, 4149, I500, 4280, G459, I64, 4349

ICD: International Classification of Diseases

### Data preprocessing

Patients who developed complications before their base visit date were excluded in this study. In this way, the study population included patients with pre-existing conditions who were at risk of developing complications only after the date of their base visit (i.e. in the subsequent 5-years).

We included only laboratory test and medication that are related to DHL conditions. These were determined a priori by clinicians managing patients with DHL, and are based on clinical practice guidelines. Additional variables, namely medication class and number of medications taken for each purpose, were derived from the individual medication data. The final list of variables in the dataset are found in Table 2. All the variables were continuous variables.

**Table 2:** List of variables (and their description) included in computing degree of similarity

No	Variables	Description
Demographic		
1	Age	Age at base visit date
Duration of disease (years)		
2	Duration of diabetes	Duration of diabetes at base visit date
3	Duration of hypertension	Duration of hypertension at base visit date
4	Duration of hyperlipidemia	Duration of hyperlipidemia at base visit date
Biomarkers		
5	Body mass index	Body mass index at base visit
6	HbA1c^a^ level (%)	Hemoglobin A1c level at base visit date
7	Systolic BP^b^ (mmHg)	Systolic blood pressure at base visit date
8	Diastolic BP^b^ (mmHg)	Diastolic blood pressure at base visit date
9	LDL^c^ level (mmol/L)	Low-density lipoprotein level at base visit date
10	HDL^d^ level (mmol/L)	High-density lipoprotein level at base visit date
11	TG^e^ level (mmol/L)	Triglyceride level at base visit date
Anti-diabetic medications: daily dose		
12	Metformin	Total daily dose of each anti-diabetic medication at base visit
13	Glipizide	
14	Gliclazide	
15	Tolbutamide	
16	Acarbose	
17	Sitagliptin	
18	Linagliptin	
19	Dapagliflozin	
20	Empagliflozin	
21	Rapid-acting insulin	
22	Isophane insulin	
23	Insulin glargine	
24	Insulin detemir	
25	Pre-mixed insulin	
Anti-hypertensive medications: daily dose		
26	Candesartan	Total daily dose of each anti-hypertensive medication at base visit
27	Captopril	
28	Enalapril	
29	Lisinopril	
30	Losartan	
31	Perindopril	
32	Telmisartan	
33	Valsartan	
34	Atenolol	
35	Bisoprolol	
36	Propranolol	
37	Amlodipine	
38	Nifedipine	
39	Hydrochlorothiazide	
40	Indapamide	
41	Spironolactone	
42	Hydralazine	
43	Methyldopa	
44	Amiloride	
Lipid-lowering medications: daily dose		
45	Lovastatin	Total daily dose of each lipid-lowering medication at base visit
46	Pravastatin	
47	Simvastatin	
48	Atorvastatin	
49	Rosuvastatin	
50	Fenofibrate	
51	Gemfibrozil	
52	Ezetimibe	
53	Cholestyramine	
Anti-diabetic medication class (count)		
54	Biguanides	Count of number of medications in each class at base visit ^f^
55	Sulphonylureas	
56	Alpha-glucosidase inhibitors	
57	Dipeptidyl peptidase 4 inhibitors	
58	Sodium-glucose co-transporter 2 inhibitors	
59	Insulin	
Anti-hypertensive medication class (count)		
60	Angiotensin-converting enzyme inhibitors and Angiotensin II receptor blockers	Count of number of medications in each class at base visit ^f^
61	Beta blockers	
62	Calcium channel blockers	
63	Diuretics	
64	Other anti-hypertensive classes	
Anti-hypertensive medication class (count)		
65	Statins	Count of number of medications in each class at base visit ^f^
66	Other lipid-lowering medications	
Medication purpose (count)		
67	Anti-diabetic medications	Count of number of medications for each condition at base visit
68	Anti-hypertensive medications	
69	Lipid-lowering medications	

^a^ HbA1c: Hemoglobin A1c

^b^ BP: Blood pressure

^c^ LDL: Low-density lipoprotein

^d^ HDL: High-density lipoprotein

^e^ TG: Triglyceride

^f^ For these variables, the count is either 0 or 1

Missing data was handled by data imputation. For non-medication variables, normal values were imputed except for age and body mass index which we used mean values. Table 3 shows the normal values imputed for the missing data. For the medications, a zero value was imputed for medications the patient was not taking.

**Table 3:** Normal values imputed for the missing data

Variable	Imputed value
Age (years)	63.2*
Body mass index (kg/m^2^)	25.2*
Systolic blood pressure (mmHg)	129.8*
Diastolic blood pressure (mmHg)	70.6*
HbA1c^a^ (mmol/L)	6.0
LDL^b^ (mmol/L)	3.0
HDL^c^ (mmol/L)	1.0
TG^d^ (mmol/L)	1.7

^a^ HbA1c: Hemoglobin A1c

^b^ LDL: Low-density lipoprotein

^c^ HDL: High-density lipoprotein

^d^ TG: Triglyceride

^*^ Mean value imputed

### Patient similarity model development

This study aimed to demonstrate that patient similarity can be used to develop an effective model for risk prediction. As such, we computed and aggregated the risk of K similar patients where K was determined using a grid-search. Min–max scaling was applied to each of the discrete variables. Additionally, expert input was also incorporated into the model, by obtaining weightage based on importance of each of the variables from consensus among a team of three clinicians. The weights were on a scale of 1 to 10 (1-least important, 10-most important). The expert consensus derived weights used in the model are shown in Table 4. The reason we elected to use a manual approach to deriving the weight was firstly to demonstrate how expert inputs can be incorporated into this approach of model development, and secondly to keep the model simple and easily explainable in how it derives the outputs (i.e. less numbers with many decimal points).

**Table 4:** Variable importance weights derived from expert consensus

Variable	Importance weight (1-least important, to 10-most important)
Age	5
Number of years with condition (Diabetes, Hypertension, Hyperlipidemia)	10
Body mass index	2
HbA1c^a^	5
Blood pressure values (Systolic and diastolic)	2.5
Cholesterol biomarkers (LDL^b^, HDL^c^, TG^d^)	1.5
Individual medication daily dose	1
Count of medications in each medication class	2
Count of medications for each condition	5

^a^ HbA1c: Hemoglobin A1c

^b^ LDL: Low-density lipoprotein

^c^ HDL: High-density lipoprotein

^d^ TG: Triglyceride

The distance metric used in K-nearest neighbour (KNN) is euclidean distance. Euclidean distance metric is a widely used distance measure for similarity search. Smaller distance implies higher degree of similarity. In this study, we present each patient in a vector of m-dimensional feature space. Accordingly, patient A is represented as A = $\left( {f_{{a_{1} }} ,~f_{{a_{2} }} ,~ \ldots f_{{a_{m} }} } \right)$ and patient B as B = $\left( {f_{{b_{1} }} ,~f_{{b_{2} }} ,~ \ldots f_{{b_{m} }} } \right)$.

Mathematically, formula for the patient similarity model which uses a weighted Euclidean distance is expressed as follows:

$$dist\left( {A,~B} \right) = \sqrt {\mathop \sum \limits_{{i = 1}}^{m} \left( {\left( {f_{{a_{i} }} *w_{i} ) - (f_{{b_{i} }} *w_{i} } \right)} \right)^{2} } ~$$

where $f_{{a_{i} }}$ and $f_{{b_{i} }}$ are the normalized i^th^ feature of patient A and patient B; m is total number of features; $w_{i}$ denotes the feature importance weights derived from expert consensus in Table 3. To ensure that no one feature dominates the distance function, the variables were normalized using minimum–maximum (MinMax) scaler with the formula as follows: $f_{{i_{{scaled}} }} = ~\frac{{f_{i} ~ - {\text{~}}f_{{i_{{min}} }} }}{{f_{{i_{{max~}} - }} ~f_{{i_{{min}} }} }}$ where $f_{i}$ is original value, $f_{{i_{{scaled}} }}$ is transformed value, $f_{{i_{{min}} }}$ and $f_{{max}}$ are the minimum and maximum values in feature $i$. The scaled data will range between values of 0 to 1. In this formula, the Diabetes, Hypertension and Lipid status (and duration of disease) were treated as independent input variables to the model. The hyperparameters used in the patient similarity model are shown in Table 5.

**Table 5:** Hyperparameters used in the final patient similarity model

Hyperparameter	Value
Nearest neighbours	10
Weights	Uniform
Metric	Euclidean distance
Search algorithm	Ball tree

The patient similarity model was compared to other methods, namely logistic regression, random forest (RF) and support vector machines (SVM). They were compared using the area under receiver operating characteristic curve (AUROC) to evaluate their effectiveness in predicting DHL complications on the same dataset. A 7:3 train-test split was used for each model development and validation, with the same random seed for all methods.

All computations and analyses were conducted using open source software machine learning libraries and packages in Python 3.7 environment. To calculate the 95% confidence intervals for the AUROC values, we additionally used the pROC package in R. The specific function uses 2000 bootstraps to perform the 95% confidence interval computation.

To demonstrate how the model generated its predictions and how the predictions can be made explainable and interpretable, a prototype system was developed to allow deployment of the patient similarity model on the full dataset to identify similar patients and to produce risk predictions for new patients not in the dataset. The prototype was packaged as a web application using the Flask framework. It was deployed as a standalone system (disconnected from the electronic medical record system).

## Results

A total of 16,144 unique patients who visited the polyclinic for DHL between April 1, 2014 and March 31, 2015 was initially included in the dataset. 6,085 of them developed any one of the complications prior to the base visit date and were removed from the final dataset. The characteristics of the 10,059 remaining patients used in study are presented in Table 6.

**Table 6:** Baseline characteristics of study patients

	n = 10,059	Missing, n (%)
Characteristics		
Age (years), mean (SD)	63.2 (11.3)	0 (0.0)
Sex, males, n (%)	4131 (41.1)	0 (0.0)
Race, n (%)		0 (0.0)
Chinese	8455 (84.1)	
Malay	635 (6.3)	
Indian	532 (5.3)	
Others	437 (4.3)	
Body mass index (kg/m^2^), mean (SD)	25.2 (4.5)	1433 (14.2)
Systolic BP (mmHg), mean (SD)	129.8 (17.7)	60 (0.6)
Diastolic BP (mmHg), mean (SD)	70.6 (10.8)	60 (0.6)
HbA1c^a^ (%)	7.1 (1.4)	7712 (76.6)*
LDL^b^ (mmol/L)	3.1 (0.9)	2175 (21.6)#
HDL^c^ (mmol/L)	1.5 (0.4)	2124 (21.1)#
TG^d^ (mmol/L)	1.4 (0.9)	2124 (21.1)
Diagnosis, n (%)		0 (0.0)
Diabetes only	150 (1.5)	
Hypertension only	1501 (14.9)	
Hyperlipidemia only	2223 (22.1)	
Diabetes & Hypertension	149 (1.5)	
Diabetes & Hyperlipidemia	315 (3.1)	
Hypertension & Hyperipidemia	4133 (41.1)	
Diabetes, Hypertension & Hyperlipidemia	1588 (15.8)	
Complications, n (%)		Not applicable^@^
Eye, n (%)	1180 (11.7)	
Foot, n (%)	117 (1.2)	
Kidney, n (%)	811 (8.1)	
Macrovascular, n (%)	1119 (11.1)	
Any of the above, n (%)	2590 (25.7)	
Look-back duration (years), mean (SD)	4.1 (1.3)	Not applicable^@^
Look-forward duration (years), mean (SD)	4.1 (1.5)	Not applicable^@^

^a^ HbA1c: Hemoglobin A1c

^b^ LDL: Low-density lipoprotein

^c^ HDL: High-density lipoprotein

^d^ TG: Triglyceride

^*^ High number of missing values as not all patients had Diabetes to require a Hemoglobin A1c test

^#^ Discrepancy between LDL and HDL values as some patients had extremely high TG to invalidate calculated LDL

@ Not applicable as these were derived data

Patients in the dataset had a mean age of 63.2 ± 11.3 years with a higher proportion of females (59.9%). The cohort also had a bias towards the combination of Hypertension and Hyperlipidemia (41.1%). The second most prevalent condition among the cohort of patient is Hyperlipidemia (22.1%), followed by the Diabetes, Hypertension and Hyperlipidemia combination (15.8%). A total of 2,509 (25.7%) patients in this study cohort developed at least one complication within five years after the base visit, with eye complications (11.7%) being the most common type.

With an initial K value of 5, the patient similarity model achieved an AUROC of 0.688 (0.667 to 0.709) in predicting DHL complications. The grid search (sensitivity analysis) yielded the best K value of 10, and the patient similarity model achieved an AUROC of 0.718 (0.697 to 0.739) (see Table 7). Compared with the other models, the patient similarity-based model was shown to be more accurate than logistic regression (AUROC = 0.695), and slightly less accurate as the SVM (AUROC = 0.766) and RF (AUROC = 0.764) models.

**Table 7:** Comparison of patient similarity model performance with other models

Model	AUROC (95% CI)
Patient similarity (K = 10)—weighted	0.718 (0.697 to 0.739)
Patient similarity (K = 10)—unweighted	0.688 (0.667 to 0.709)
Logistic regression	0.695 (0.672 to 0.718)
Random forest	0.764 (0.744 to 0.784)
Support vector machine (kernel = linear)	0.766 (0.746 to 0.785)

With regard to the clinician assigned weights, we found that it helped to improve the model performance. When removing the variable importance weights shown in Table 3, the patient similarity model had a poorer performance (AUROC = 0.688). While the coefficient values or Gini importances of individual medications was similarly low across all the two models, which corresponds with the expert consensus for the patient similarity model, there were differences in the way the variables were ranked between the models. The logistic regression coefficients and RF Gini importances can be found in the Additional file 1: Table 1.

### Patient similarity model explainability and interpretability

**Fig. 1:**
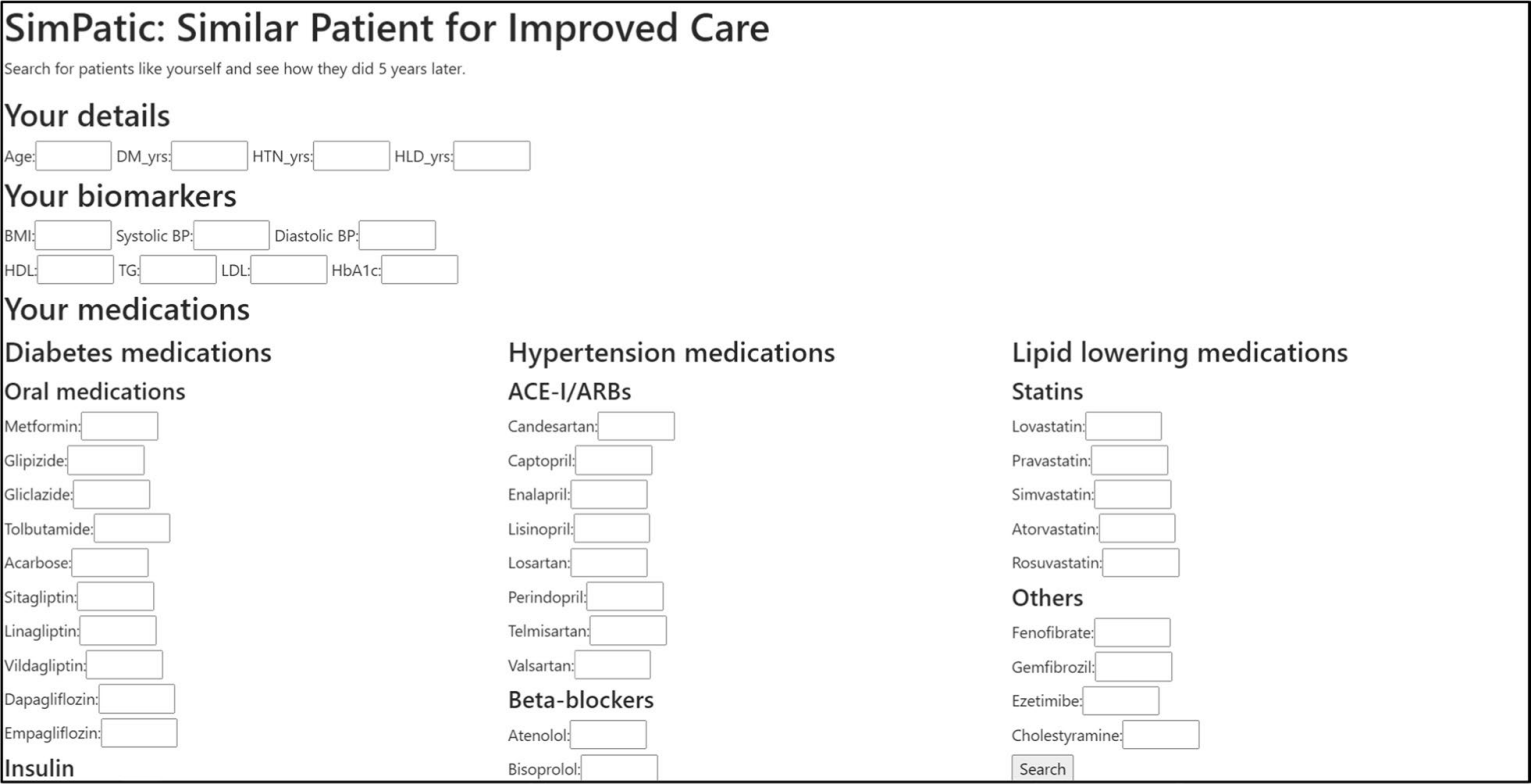
The landing page (zoomed in at 175%) of the prototype web application using the patient similarity model. Users can enter demographic, biomarker and medication inputs to identify similar patients from the database

The patient similarity model was implemented as a web application to allow users to enter details about a new patient and to generate an estimated risk of DHL complications (see Fig. 1).

In terms of explainability, this approach is transparent in how it generates its risk predictions. The first step is to perform a multi-dimensional search across 69 variables, with importance weights applied, to find the ten most similar patients, based on Euclidean distance. The next step is to then aggregate the known outcomes of these ten patients from the database to compute the risk. For example, if four out of the ten patients had a DHL complication, the estimated risk for the new patient would be 40%.

In terms of interpretability, for the same example above, the predicted risk can be understood by patients as “based on the ten most similar patients to myself, four in ten of them had a DHL complication within the next 5 years”. Furthermore, with the ability to pinpoint the ten most similar patients, healthcare providers can select a particular similar patient to view his/her longitudinal medical history over the subsequent five years. This could be used as a basis for crafting a more compelling narrative to deliver prognostic information.

### Proof of concept

**Fig. 2:**
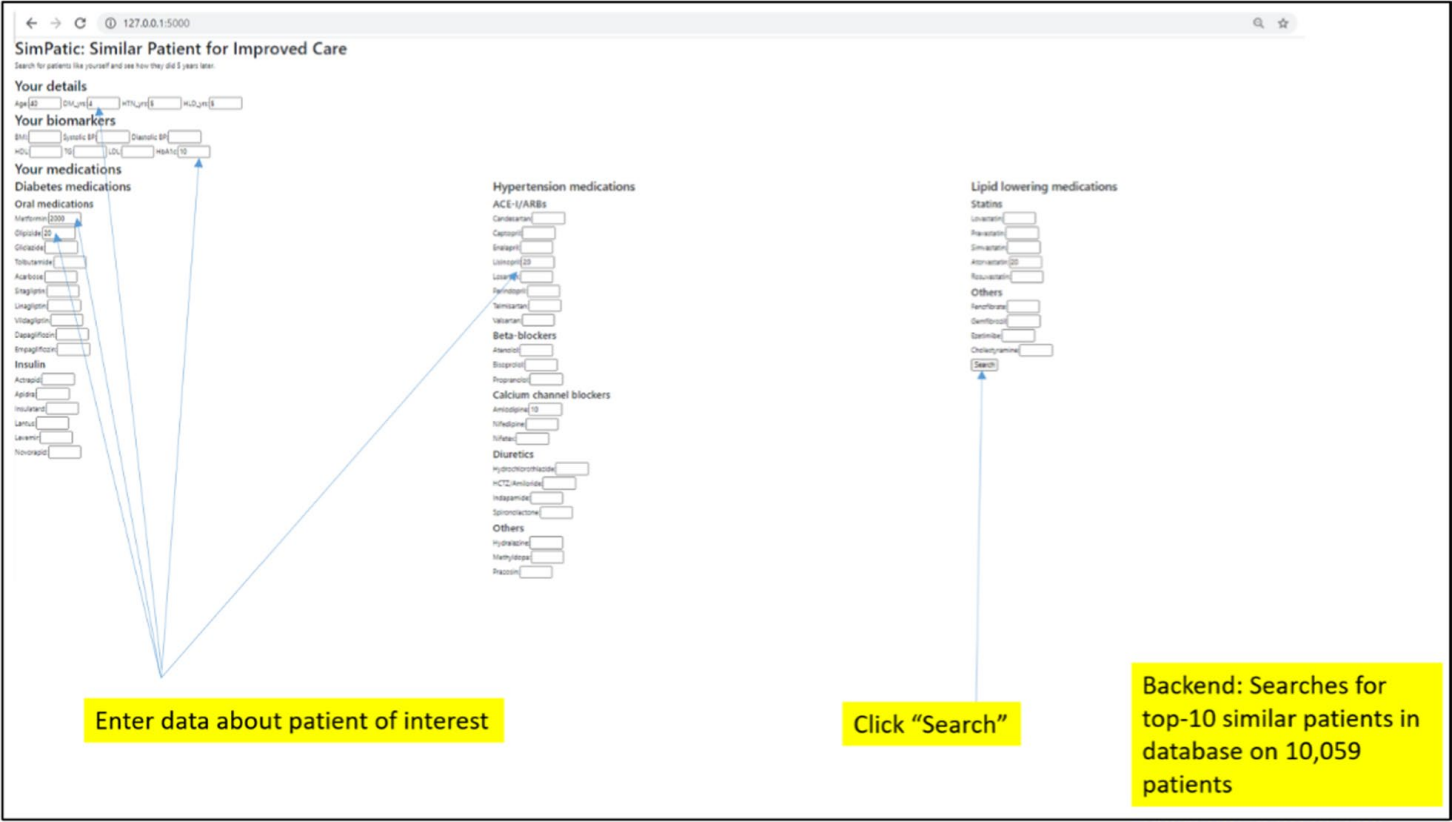
Data input into the prototype web application. The attending doctor enters the details of Patient X into the web application. Fields are non-mandatory. After entering the details, the attending doctor clicks the “Search” button which triggers the patient similarity model to identify the top-10 most similar patients in the database

To illustrate how the web application can be used, we conducted mock consultation with a young patient with poorly controlled diabetes (Patient X). The attending doctor (primary user of the application) entered relevant details of Patient X in the web application. Patient X was 40 years old with pre-existing Diabetes, Hypertension and Hyperlipidemia for 4 years, 5 years and 5 years respectively. He had poorly controlled Diabetes with HbA1c of 10.0%. He was taking metformin (total daily dose [TDD]: 2000 mg), and glipizide (TDD: 20 mg), lisinopril (TDD: 20 mg), amlodipine (TDD: 10 mg) and atorvastatin (TDD: 20 mg) (see Fig. 2).

**Fig. 3:**
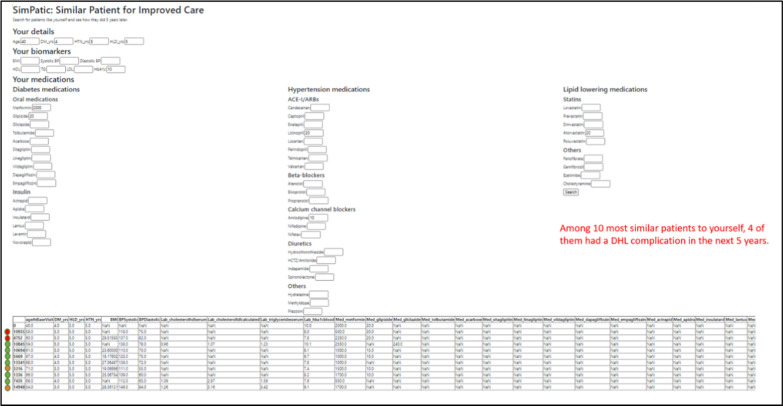
An anonymized list of the top-10 most similar patients to Patient X is presented. An aggregate prognostic value is calculated based on the proportion of the top-10 patients who encountered a DHL complication. The green/orange/red indicators represent the outcomes of each patient over the subsequent 5 years from base visit. Green indicates that the patient did well (i.e. no complications). Orange indicates the patient had some complications or worsening in biomarker, while red indicates that the patient did poorly with multiple complications. In this case, four of the ten patients had either orange or red indicators

The backend system would identify the top-10 most similar patients from the database of 10,059 patients and display them as a list of anonymised records (see Fig. 3). In this case, among the top-10 most similar patients to Patient X, four of them had developed a complication. This can be interpreted by Patient X to be “for the 10 most similar patients to myself, four had a DHL complication in the next five years.” The attending doctor would leverage on such prognostic information to prompt Patient X to take action to optimize his/her glycemic control.

**Fig. 4:**
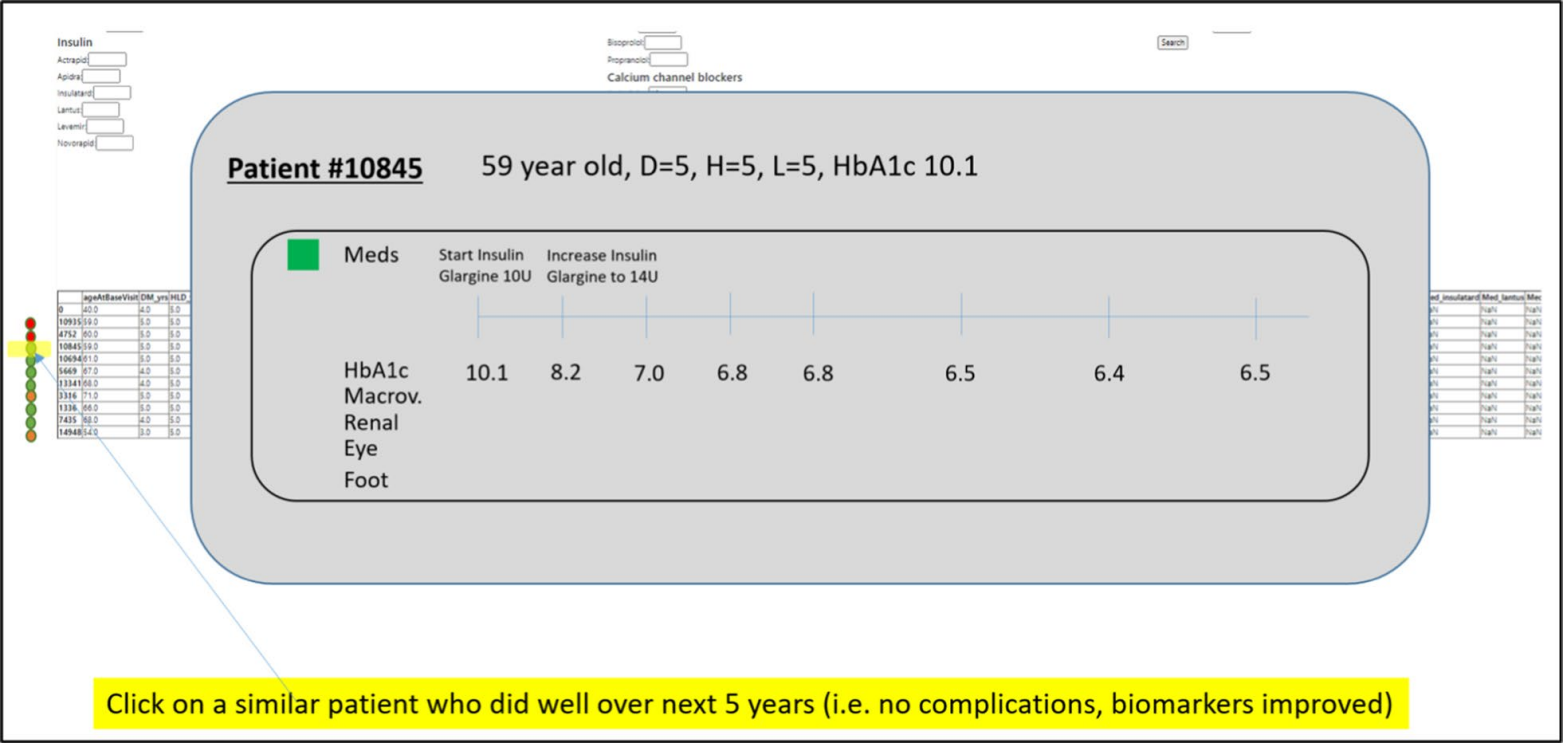
Timeline of a similar patient (Patient #10845). A particular similar patient can be selected to produce a timeline. In this case, Patient #10845 was selected to illustrate to Patient X a patient like himself who did well, and what Patient #10845 did to achieve the good results

Going one step further, the system also allows the attending doctor to select a particular similar patient to generate a timeline. In this case, the attending doctor selects Patient #10,845 who is a 59 year old with Diabetes, Hypertension and Hyperlipidemia each for 5 years. Patient #10,845 also has poorly controlled Diabetes with HbA1c of 10.1%. From the timeline, it shows Patient #10,845 starting Insulin Glargine and later increasing the dose of the medication to eventually achieve good glycemic control and staved off all complications (see Fig. 4). Using this timeline information, the attending doctor would be able to craft a case-based narrative to recommend Patient X to start Insulin Glargine to achieve glycemic control. Conversely, the attending doctor can select a patient, who has developed a complication, to present an adverse scenario to alert Patient X.

## Discussion

In this study, we presented an approach of using a conventional machine learning technique, namely K-nearest neighbor, which can incorporate expert clinician knowledge to develop a patient similarity model for DHL complications prediction. For usage of the model, we proposed a two-layered presentation of information to patients. The first layer presents an aggregated risk of similar patients, while the second layer presents a narrative of a similar patient. The latter addresses a gap in conventional risk models, such as logistic regression models. Furthermore, unlike conventional risk models which provide more abstract predictions (i.e. “this is your estimated risk”), patient similarity models can couch the informational output more realistically (i.e. “this is what actually happened to X out of Y patients similar to yourself”). Given that different doctors and patients have unique preferences for the type and style of information delivery, patient similarity models will probably not replace conventional risk models, but complement them with a new variety of information that can be selectively used in the appropriate consultation context.[28].

Previous work had employed different strategies to develop explainable prediction models [9, 16–19]. Shickel et al. used a self-attention approach to highlight time steps in their model’s input time series that the model believes to be most important in formulating the final mortality prediction. This was visualized in a two-dimensional grid [16]. Zhang et al. also developed an attention based prediction model and used a heatmap to present the relative importance of events over time [17]. While Rajkomar et al. explored using free text data within the dataset to enhance explainability, Lundberg et al. presented several tools like dependence plots and explanation embeddings to better explain tree-based model outputs [18, 19].

In spite of these developments, adopting them in clinical settings remains a challenge. Our patient similarity approach is easy to use and may be applied to various settings, diseases and patient groups. As long as there is an available database of patient records, a patient similarity CRPMs can be developed. Using this approach, they can be contextualized to the local patient characteristics and type of data variables in the database, which can then be used to develop an end-product that is locally relevant and applicable.

Complementing hard facts with patient stories have been shown to be an effective means of patient education by increasing personal relevance and reducing counter-arguing [29]. Bokhour et al. showed that an education intervention using patients’ success stories in controlling their hypertension resulted in more emotional engagement and reported intentions to change behavior [30]. This is further supported by Lesselroth and Monkman who have advocated embedding powerful narratives and stories in health information technology and for further research and development to evaluate its effectiveness [31]. In this way, our idea of using similar patients to craft narratives for CRPMs is an elegant way of weaving together qualitative and quantitative prognostic information to support decision-making.

While our experimental patient similarity model may not have the best performance in terms of discriminatory power, it was able to achieve an acceptable AUROC comparable to other machine learning methods such as SVM and RF [32]. With fine-tuning of other hyperparameters and ongoing research into novel similarity metrics and algorithms, patient similarity models may be able to perform even better in future [24]. For now, there is a trade-off between accuracy and explainability. In addition, unlike other CRPMs which generate a probabilistic output for a particular patient, the patient similarity model risk estimates are interpreted on the basis of “what actually happened to patients like yourself”, rather than “what will happen to you”. Based on this perspective, we posit that the validation of patient similarity models may not need to be as heavily scrutinized as other types of CRPMs before deployment.

We acknowledge that there are several limitations in this current patient similarity model. Firstly, it does not use all the variables, such as gender, race, diet and lifestyle, which are associated with DHL complications. The reason we did not include diet and lifestyle was because these data were not available in the datasets. While gender and race are used in some clinical risk scores, they were not included in our patient similarity model as their associations with DHL complications are comparatively weak.[33, 34] Furthermore, although we had different races in our study populations, they were mostly South Asian. This ethnic homogeneity would have lessened the value of including the race variable. If the variables were not used in the patient similarity model, they were also not used in all the other models used for comparison. Secondly, the current patient similarity model uses input data from a single time point. This would not completely be reflective of DHL which are chronic diseases. While we have included the duration of these diseases, plotting out the trajectories of these would be even more useful. Such disease progression models have been shown to be effective at predicting cardiovascular risk of diabetes and lipid disorders.[35, 36] These progression models or trajectories can be incorporated as additional input variables to improve model performance further. Thirdly, because the model building and validation included right-censored cases, it could have introduced bias. This is probably not very significant in our case since majority of the cases were not censored, but approaches such as inverse probability of censoring weights should be considering in datasets where larger proportion of censored cases are used.[37] Fourthly, such an approach of using K-nearest neighbour would rely on having a database of patient information to search for similar patients. This may require regular updates to the database, requiring additional effort to maintain. Furthermore, this may also encounter some data security concerns, which may be alleviated by removing personally identifiable information from the database. Lastly, the interpretability of the outputs would depend on the complexity of the cases and the size of the data. For example, in some cases this approach may not be able to find sufficient number of similar patients, by the fact that the case of interest is an outlier. In this case, the model should flag that it is not applicable. This would be similar to other models which do not perform well on outlier type of cases. However these could potentially be addressed in this approach by having an as large as possible dataset, and with as much details, to perform the search on.

Looking ahead, patient similarity analytics can be used to develop effective, explainable and interpretable CRPMs as clinical decision support and shared-decision making tools to enhance patient care. The patient similarity model will be fine-tuned and optimized with research to create optimal training hyperparameters, including search algorithms and similarity metrics. We will also explore other methods of deriving feature weights such as multivariate feature selection and Mahalanobis distance with a trainable covariance weight matrix and other machine learning model training. We will assess a to-be-developed patient similarity based tool for clinical decision support in clinical practice. For this, we plan to conduct a multi-site hybrid implementation trial to determine its impact on decision-making quality, patient and clinician satisfaction, patient health outcomes and process outcomes (such as consultation duration). Ultimately, we look forward to the tool being integrated within our electronic medical records and other IT systems, and clinical workflows.

## Conclusion

In this study, we have presented patient similarity as an approach to develop an explainable and interpretable CRPM. The patient similarity model is comparable to other machine learning based models in predicting DHL-related complications. Furthermore, we introduced a prototype system to demonstrate transparency in the prediction process and the utility of the generated results to craft patient narratives. A proof of concept illustrates how this can be used in clinical practice. Adopting a patient similarity approach in developing CRPM can result in the development of more explainable and interpretable clinical decision support tools to ultimately enhance the decision-making process in clinical practice.

## Supplementary Information

**Additional file 1.** Comparison of variable weights between logistic regression, random forest and those derived from expert consensus methods.

## Data Availability

The datasets analyzed during the current study are not publicly available as they contain information that are sensitive to the institution. They may be made available from the corresponding author on reasonable request.

## Abbreviations

AUROC: Area under the receiver operating characteristic curve
BP: Blood pressure
CKD-EPI: Chronic kidney disease epidemiology collaboration
CRPM: Clinical risk prediction models
DHL: Diabetes, hypertension and Hyperlipidemia
EMR: Electronic medical records
HbA1c: Glycosylated haemoglobin
HDL: High-density lipoprotein
ICD: International classification of diseases
KNN: K-nearest neighbour
LDL: Low-density lipoprotein
RF: Random forest
SVM: Support vector machines
TDD: Total daily dose
TG: Triglyceride
