# Supplementary material for: Patient similarity analytics for explainable clinical risk prediction
Source: BMC Med Inform Decis Mak. 2021 Jul 1;21:207. doi: 10.1186/s12911-021-01566-y (PMC8247104; doi:10.1186/s12911-021-01566-y)
Supplement: Supplementary file 1 — Additional file 1. Comparison of variable weights between logistic regression, random forest and those derived from expert consensus methods. [file 12911_2021_1566_MOESM1_ESM.docx]

Table 1. Logistic regression model coefficients and random forest Gini importance, compared with importance weights derived from expert consensus. To note that the variable weights are relative to weights of the other variables from the same method of derivation.

| No. | Variables | Logistic regression coefficients | Random forest Gini importance | Importance weights derived from expert consensus |
| --- | --- | --- | --- | --- |
| 1 | Age | 0.003245 | 0.087644 | 5 |
| 2 | Duration of Diabetes | 0.022735 | 0.028251 | 10 |
| 3 | Duration of Hypertension | -0.00856 | 0.028916 | 10 |
| 4 | Duration of Hyperlipidemia | -0.04711 | 0.029901 | 10 |
| 5 | Body mass index | -0.02693 | 0.075355 | 2 |
| 6 | HbA1c^a^ level (%) | -0.00687 | 0.066198 | 5 |
| 7 | Systolic BP^b^ (mmHg) | 0.00061 | 0.073132 | 2.5 |
| 8 | Diastolic BP^b^ (mmHg) | -0.01383 | 0.068713 | 2.5 |
| 9 | LDL^c^ level (mmol/L) | -0.02255 | 0.067141 | 1.5 |
| 10 | HDL^d^ level (mmol/L) | -0.01952 | 0.069241 | 1.5 |
| 11 | TG^e^ level (mmol/L) | 0.00723 | 0.064587 | 1.5 |
| 12 | Metformin | 0.000497 | 0.019572 | 1 |
| 13 | Glipizide | 0.008469 | 0.006728 | 1 |
| 14 | Gliclazide | 0.003336 | 0.004842 | 1 |
| 15 | Tolbutamide | 1.30E-05 | 0.002498 | 1 |
| 16 | Acarbose | -0.00313 | 0.001476 | 1 |
| 17 | Sitagliptin | 0.000169 | 0.002308 | 1 |
| 18 | Linagliptin | -5.63E-05 | 4.40E-05 | 1 |
| 19 | Dapagliflozin | 0.000589 | 3.80E-05 | 1 |
| 20 | Empagliflozin | 0 | 0 | 1 |
| 21 | Rapid-acting insulin | -4.27E-05 | 0.000229 | 1 |
| 22 | Isophane insulin | 0.001289 | 0.000303 | 1 |
| 23 | Insulin glargine | 0.003464 | 0.000663 | 1 |
| 24 | Insulin detemir | 0.003302 | 0.000259 | 1 |
| 25 | Pre-mixed insulin | -0.00154 | 0.001039 | 1 |
| 26 | Candesartan | 0.00239 | 0.000625 | 1 |
| 27 | Captopril | 0.008831 | 0.000232 | 1 |
| 28 | Enalapril | 0.029418 | 0.010806 | 1 |
| 29 | Lisinopril | 0.019989 | 0.008501 | 1 |
| 30 | Losartan | 0.008153 | 0.011121 | 1 |
| 31 | Perindopril | 0.00114 | 0.001006 | 1 |
| 32 | Telmisartan | 0.001191 | 0.004401 | 1 |
| 33 | Valsartan | 0.005961 | 0.003604 | 1 |
| 34 | Atenolol | 0.00268 | 0.012543 | 1 |
| 35 | Bisoprolol | 0.009002 | 0.008026 | 1 |
| 36 | Propranolol | 0.007247 | 0.00072 | 1 |
| 37 | Amlodipine | 0.028259 | 0.021462 | 1 |
| 38 | Nifedipine | 0.001968 | 0.00912 | 1 |
| 39 | Hydrochlorothiazide | 0.014405 | 0.005025 | 1 |
| 40 | Indapamide | -4.96E-05 | 8.67E-05 | 1 |
| 41 | Spironolactone | 0.007024 | 0.000494 | 1 |
| 42 | Hydralazine | 0.005536 | 7.55E-05 | 1 |
| 43 | Methyldopa | 0.001505 | 0.000737 | 1 |
| 44 | Amiloride | 0 | 0 | 1 |
| 45 | Lovastatin | -0.00287 | 0.004952 | 1 |
| 46 | Pravastatin | 0.005706 | 0.001364 | 1 |
| 47 | Simvastatin | 0.019916 | 0.024498 | 1 |
| 48 | Atorvastatin | 0.047767 | 0.012748 | 1 |
| 49 | Rosuvastatin | 0.012043 | 0.00278 | 1 |
| 50 | Fenofibrate | 0.000582 | 0.006451 | 1 |
| 51 | Gemfibrozil | -0.00146 | 0.000229 | 1 |
| 52 | Ezetimibe | 0.004254 | 0.001718 | 1 |
| 53 | Cholestyramine | -5.73E-05 | 0.000369 | 1 |
| 54 | Biguanides | 0.005377 | 0.014052 | 2 |
| 55 | Sulphonylureas | 0.002568 | 0.007808 | 2 |
| 56 | Alpha-glucosidase inhibitors | 1.97E-05 | 0.000951 | 2 |
| 57 | Dipeptidyl peptidase 4 inhibitors | -4.92E-05 | 0.001838 | 2 |
| 58 | Sodium-glucose co-transporter 2 inhibitors | 5.89E-05 | 5.04E-05 | 2 |
| 59 | Insulin | 0.00111 | 0.002089 | 2 |
| 60 | Angiotensin-converting enzyme inhibitors and Angiotensin II receptor blockers | 0.007693 | 0.012806 | 2 |
| 61 | Beta blockers | 0.003467 | 0.008116 | 2 |
| 62 | Calcium channel blockers | 0.002644 | 0.00954 | 2 |
| 63 | Diuretics | 0.001178 | 0.004064 | 2 |
| 64 | Other anti-hypertensive classes | -5.57E-05 | 0.000794 | 2 |
| 65 | Statins | -0.0005 | 0.008084 | 2 |
| 66 | Other lipid-lowering medications | 0.001018 | 0.004591 | 2 |
| 67 | Anti-diabetic medications | 0.009085 | 0.02165 | 5 |
| 68 | Anti-hypertensive medications | 0.014926 | 0.021519 | 5 |
| 69 | Lipid-lowering medications | -0.00051 | 0.013375 | 5 |

^a^ HbA1c: Hemoglobin A1c.

^b^ BP: Blood pressure.

^c^ LDL: Low-density lipoprotein.

^d^ HDL: High-density lipoprotein.

^e^ TG: Triglyceride.

^f^ For these variables, the count is either 0 or 1.
